# Experiments in Computing: A Survey

**DOI:** 10.1155/2014/549398

**Published:** 2014-02-04

**Authors:** Matti Tedre, Nella Moisseinen

**Affiliations:** ^1^Department of Computer and Systems Sciences, Stockholm University, 16440 Kista, Sweden; ^2^Faculty of Behavioural Sciences, University of Helsinki, 00014 Helsinki, Finland

## Abstract

Experiments play a central role in science. The role of experiments in computing is, however, unclear. Questions about the relevance of experiments in computing attracted little attention until the 1980s. As the discipline then saw a push towards experimental computer science, a variety of technically, theoretically, and empirically oriented views on experiments emerged. As a consequence of those debates, today's computing fields use experiments and experiment terminology in a variety of ways. This paper analyzes experimentation debates in computing. It presents five ways in which debaters have conceptualized experiments in computing: feasibility experiment, trial experiment, field experiment, comparison experiment, and controlled experiment. This paper has three aims: to clarify experiment terminology in computing; to contribute to disciplinary self-understanding of computing; and, due to computing's centrality in other fields, to promote understanding of experiments in modern science in general.

## 1. Introduction

After the birth of the stored-program paradigm in the mid-1940s, computing as a discipline started to form up. The first step in the discipline creation was to separate it from the fields that gave birth to it, especially from mathematics and electrical engineering. In the 1960s and the 1970s the field was divided over a debate concerning the mathematical nature of computing (e.g., [1–6]). There were a variety of formal, theory-oriented views of computing as a discipline. Some theoretically proficient computer scientists emphasized the mathematical analysis of algorithms for the general conclusions such analysis could provide [7–12]. Another group focused on developing a mathematical theory of program construction [13–18]. The most vehement advocates of a mathematical theory of computing went as far as to suggest that programming as an activity is fully reducible to mathematics [19]. In the theoretical advocates' visions of the discipline, the role of empirical work and experimentation was often ambiguous, as it was rarely, if ever, discussed in detail.

Another debate that characterized the development of computing as a discipline was concerned with the field's engineering character. Engineering aspects of computing were, for several decades, effectively kept out of the academic debate about computing as a discipline; despite the fact that the first computers were built in universities, they were used for applied sciences, and the development of early computing in universities had a strong engineering character [20–23]. The late 1960s, however, saw a new turn in these debates when software engineering was brought to limelight [24]—and harshly criticized [25]. For decades, software engineering remained a target of sustained criticism. Software engineers were accused of basing their work on a combination of anecdotal evidence and human authority [26]. What is more, meta-analyses of literature found that a large portion of software engineering articles failed to experimentally validate their results [27–29]. Lacking experimentation was one of the commonly criticized aspects of software engineering.

A third debate about the essence of computing as a discipline was concerned with the scientific character of computing. There were arguments over whether computing is a science or not, and there were arguments over what might computing be a science of [30]. In one of the influential early defenses of the scientific nature of computer science it was argued that computer science is the study of computers and phenomena surrounding them [31]. Other proposals for the subject matter of computing included, for instance, information, algorithms, classes of computations, programming, complexity, and procedures [5, 32–37].

Arguments that looked at the subject matter of computing never managed to settle the debate over the scientific character of computing. But over the course of time, the focus of the “science” debates shifted from subjects to activities. It became increasingly common to argue that computing is indeed science—not by virtue of its subject matter but by virtue of its *method of inquiry*.

The methodology question entered computing debates gradually. Many early arguments for computing as a science glossed over methodological questions. Although some descriptions of the “axiomatic” or “mathematical” sciences of computation compared computing with natural sciences (e.g., [16]), they rarely discussed either the relevance of the scientific method to computing or the role of experiments in the field. Similarly, one of the first descriptions of computing as an empirical science, by Newell et al. [31], was vague about methods and empirical approaches in the science of computing. The methodology question was finally brought into limelight by the *experimental computer science* debate, when a campaign for “rejuvenating experimental computer science” started at the turn of the 1980s [38–41].

The view that computing is an inseparable combination of three very different intellectual traditions—theory, engineering, and empirical science [42]—complicates many debates about computing. One such debate is the “experimental computer science” debate. The words “experiment” and “experimental” are understood very differently between the traditions, which makes it difficult to grasp the intent of each argument on experimental computer science. This paper presents a survey of arguments about experimental computer science and presents that at least five different uses of the terms “experiment” and “experimental” can be found in the computing literature. This paper is a survey of how terminology is *actually* used and not of how it *should* be used. For instance, in the engineering tradition experimentation terminology is used much more loosely than in the tradition of experiment-based science. In short, the paper seeks an answer to the question, “*What do computer scientists mean when they talk about experiments in computer science*?”

## 2. Experimentation in Computing

Among researchers in computing disciplines there is wide support for views of computing as an empirical or experimental science. However, the terms *empirical* and *experimental* are not always used coherently. In sciences in general, it is relatively common to see the term “empirical” used to refer to research that relies on observation-based collection of primary data. The term “empirical research” stands in contrast with theoretical and analytical research. In many fields of science the term “experimental” goes deeper than “empirical” and refers to a specific kind of research, where controlled experiments are used for testing hypotheses. However, in the field of computing the term “experimental” has been used in a much more wider range of meanings.

The role of experimentation in computing became a hot topic when Feldman and Sutherland [40] published their report entitled “Rejuvenating Experimental Computer Science.” That report recommended that universities and the U.S. government should recognize and support experimental computer science. Denning [38] joined ranks with the Feldman committee and wrote that no scientific discipline can be productive in the long term if its experimenters merely build components. Also the ACM Executive Committee, which included Denning, agreed with the Feldman committee in that experimental computer science was undervalued at the time [41].

The “rejuvenating” report marked a shift of focus in methodology debates from the roles of theory and subject matter to the amount and methodological quality of empirical work in computing. The following decades saw numerous descriptive and normative arguments on the role of empirical and experimental research in computing. While some described how computer scientists actually work, others prescribed how they should work. Several studies compared research reports in computing with those in other fields—usually natural sciences or established branches of engineering [28, 45]. In those studies, it was a common finding that research in computing fields experiment significantly less than researchers in many other disciplines do [28, 45].

Over the course of time, many authority figures in computing advised computer scientists to experiment more [46, 47]. Given that much of that encouragement was due to inspiration from other fields, it is interesting to look at the computing side of the story. In particular, what do computer scientists from different backgrounds mean by “experimental computer science?” This section presents, firstly, the context of the experimental science debate through four viewpoints: empirical dimensions of computing, subjects of experimentation, experimental activities, and various terminological and classification viewpoints. Secondly, this section outlines critical viewpoints to experiments in computing, as presented in computing literature.

### 2.1. Experimentation in Computing Context

#### 2.1.1. Empirical Dimensions of Computing

All the different accounts of experiments in computing—from controlled experiments to experimental algorithmics—fall into the broader category of *empirical* work. Computing and empirical research have been coupled in the literature in various ways, of which one particular perspective is discussed below. Computing and computers are, for one thing, *subjects* of research. Second, they are *instruments* of research. Third, they may be both at once.

One popular way of discussing computing and experimentation is to see computers and phenomena around them as a *subject of research* (e.g., [31]). There is a rich body of experimental work on computers, programming languages, interfaces, users, and algorithms, just to name a few. Some experiments are done in a very controlled manner, while some authors refer to their exploratory work as “experimental.” Viewing computing, computers, and phenomena surrounding them as a subject of inquiry opens doors for a variety of views on experimentation, and this paper looks at that aspect of experiments in computing.

Another popular way of discussing experimentation in computing is through seeing computers as *research instruments* in other fields. The history of computing and computers as instruments for experiments (simulations) in other fields is a long-established one. In his introduction to the famous 1946 Moore School lectures, Stibitz [48] argued that digital computers are an incredible laboratory where “*the ingredients of every experiment are perfectly isolated*.” Stibitz wrote that computers offer unlimited precision and an unlimited supply of instruments for research. Later on, the first modern computers were used for applied sciences, such as ballistics calculations, warfare [49, page 214], meteorology, astronomy [50, page 189], and quantum physics [25, page 122]. Progress in modern science is so heavily dependent on computing that different authors have called the increased dependence “algorithmization” of sciences [51], “the age of computer simulation” [52], and even an “info-computational” view of the world [53]. Computing has introduced a plethora of tools for other sciences—take, for instance, virtual experiments, simulations, heuristic models, and neural networks [52]. Viewing computing as an instrument of research paints another image on experimentation, different from viewing computing as a subject of research.

Various kinds of models also pervade the field of computing. One can easily consider specifications, program texts, and programming languages to be certain kinds of models [54, 55]. The experiment is a central part of validating models or testing the fit between the model and the world [56]. However, when computer models are used as tools, one should ask which discipline is actually being studied. Colburn [43] used computational models in genetics as an example. Is the programmer actually doing genetics or computer science? In many computational sciences joint work benefits both computing and the field where it is applied [57]. That is, computing can at once be a tool and a subject of study. This paper, however, does not focus on the instrumental aspect of computing but on research of computing for computing's sake.

#### 2.1.2. Subjects and Topics

Another angle at describing the context of experimentation in computing is to look at its subjects and topics. As there is already a good number of arguments about experimental computer science, one can borrow examples directly from the literature. In his discussion on experiments in computer science, Denning [39] brought up research on memory policies in time sharing and research on queuing networks. Freeman [58] proposed examples of a robot competition, research of data-intensive supercomputing, and research of future network architectures. Feldman and Sutherland [40] included advanced applications of computers. Gustedt et al. [59] highlighted research on, for instance, grid computing, parallel computing, large-scale distributed systems, and various other projects in large-scale computing. Basili and Zelkowitz [60] mentioned software engineering and high-end computing. Various authors from Chaitin to Zuse have argued that nature itself calculates [61, 62]. In the end, subject as such is not of importance [63]. Any subject can be studied scientifically, and many can be studied experimentally.

#### 2.1.3. Activities

One can also take a look at what kind of activities the term “experimental computer science” might cover. In the original “rejuvenating” report [40], experimenting in computer science was characterized as exploration (page 498), construction and testing (page 499), hypothesis-testing, demonstration, and modeling (page 500). Denning listed modeling, simulation, measurement, validation, prototyping, testing, performance analysis, and comparisons [64]. Other participants of the debate mentioned, for example, measuring, testing, making hypotheses, observing, collecting data, classifying, and sustaining or refuting hypotheses [39, 41]. As a prime example of experimental computer science, Denning [39] referred to performance analysis—the construction, validation, and empirical evaluation of computer systems. Belady [65] wrote that his experimental computer science involved building prototypes, observing, organizing observations, and formalizing them into models. All the activities above are central to science, but they are central to different kinds of science.

At the end of the 1980s the famous report “Computing as a discipline” by Denning et al. [42] raised modeling as one of the three cornerstones of computing. In that report, experiments played a role similar to their role in natural sciences. Denning et al. described the cycle of work on the science side of computing through four steps, (1) Form a hypothesis, (2) construct a model and make a prediction, (3) design an experiment and collect data, and (4) analyze results. Freeman [58] dropped the hypothesis part and advocated a view of experimentation in computing based on a cycle of observation, measurement, and analysis of results. Gelernter [66, page 44] emphasized the generalizability of results, he explicitly noted the deductive and inductive phases of research, and he argued that computing is indeed a science insofar as its combination of theoretical foundations and experiments allows the making and proving of general statements.

**Figure 1:**
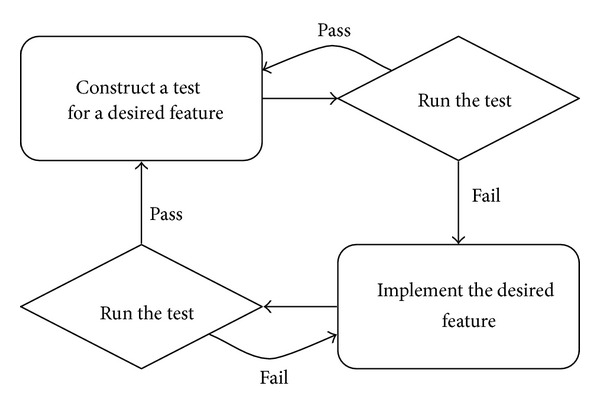
Cycle of work in test-driven development.

One unique formulation of an experiment-like procedure in computing—one with automated and repeatable experiments—can be found in the cycle of test-driven development (Figure 1; see, e.g., [67]). In test-driven development, each cycle in software construction starts with writing a test for an added software feature. The procedure continues with running all the tests and seeing the previously added test fail, writing code that implements the wanted feature, and running the tests again to see if the newly written code really implements the desired functionality. In other words, the programmer starts from a certain functionality requirement, designs an automated experiment that is aimed at testing that functionality, and implements code that passes all the new and previous tests.

In the field of software engineering there is a rich history of discussions on experimental methods—including highly influential accounts like that of Basili, Selby, and Hutchens [68]—although terminology in those discussions is often used differently from what the stalwart proponents of experimental computer science advocated. Zelkowitz and Wallace [28, 29] categorized “experimental approaches” in software engineering into three categories: *observational* methods, which collect data throughout the project; *historical* methods, which collect data from already completed projects; and *controlled* methods, which attempt to increase the statistical validity of results by providing multiple instances of observations. Of observational methods, they listed project monitoring, case study, assertion, and field study [28]. Of historical methods, they listed literature search, legacy data, lessons learned, and static analysis. Of controlled methods, they listed replicated experiment, synthetic environment experiments, dynamic analysis, and simulation. It is important to note that Zelkowitz and Wallace [28, 29] did not call their lists “empirical” but “experimental” models and approaches. They argued that their categories cover the previously presented taxonomies, such as the nine variants of quantitative and qualitative experiments described by Kitchenham [69] as well as the six types identified by Basili [70]. Again, the descriptions of experimentation in software engineering are all central to science but to different kinds of science.

On the broader level, Morrison and Snodgrass [71] wrote that debugging is one aspect of the scientific method that computer scientists do well. Different from Dijkstra [13], who opposed debugging as “*putting the cart before the horse*,” Morrison and Snodgrass described debugging as “*one of the purest forms of empirical investigation*.” There are indeed various attempts to describe debugging as a “science of debugging” [44, 72, 73]. One of the pioneering works in the philosophy of experiment, by Hacking [74], named “debugging” as a central element in modern experimentation—although its meaning in the context that Hacking discussed is different from its meaning in computing. Also other modern views of the scientific method include debugging, under different names, in the cycle of scientific research (e.g., [75]). The literature on the philosophy of engineering takes that aspect of research further through, for instance, *parameter variation*: the repeated measurement of a device's performance, while systematically adjusting the device's parameters of its conditions of operation [76, page 139].

Colburn [43] sketched another formulation of experiment-based work in computer science in the form of “solution engineering.” In various branches of computer science the usual scenario includes rigorous requirements, and the task of the computer scientist is to engineer an algorithmic solution.  Table 1 presents Bartley's [44] description of debugging, in parallel with Colburn's [43] “solution engineering” and a simplified three-step view of the scientific method.

In Colburn's analogy in Table 1, what is being tested in the scientific method is not the experiment but the hypothesis. The experiment is a tool for testing the hypothesis. Similarly, in Colburn's analogy, what is being tested in problem solving in computer science is not the program but the algorithm. The program is written in order to test the algorithm. In this analogy, writing a program is analogous to constructing a test situation. Khalil and Levy [35] made a similar analogy as they wrote, “*programming is to computer science what the laboratory is to the physical sciences*.”

Although solution engineering presents another view of experimentation in computing disciplines, it has been argued that an experiment in science can never test an isolated hypothesis but the whole theoretical group: assumptions, auxiliary hypotheses, and indeed the whole test situation [77, 78]. Similarly, running a program cannot accept or reject an algorithm alone, but it can only accept or reject the whole test system—including, for example, the operating system, hardware, quality of data, and contingent environmental inference. It can never be ruled out that the algorithm and the corresponding program were fine but something else in the test system caused wrong results—and it can not be ruled out that the program was incorrect but, due to a problem with the test system, it contingently yielded right results.

#### 2.1.4. Terminology and Classifications

There have also been analyses of experimentation terminology in computing. Feitelson [79] distinguished between three uses of the term “experimental computer science.” He argued that the most prominent use of the term is to use it as a counterpart to theoretical computer science. The second use of the term, according to Feitelson [79], is as a part of a feedback loop for the development of models, systems, and various other elements of computer science. Feitelson's third notion referred to the adoption of scientific experimental methods for the evaluation of computer systems. Gustedt, Jeannot, and Quinson presented four examples from large-scale systems: *in situ* experiments, emulation, benchmarking, and simulation [59].

Amigoni et al. [80] analyzed experimental activities in mobile robotics and classified them according to their purposes, the data sets they employ, and their measured quantities, be they intrinsic or extrinsic. Regarding *purposes*, they found demonstrations, gathering insight into a system's behavior, assessing limits of applicability, and comparing systems. Regarding *data sets*, they found publicly available instances, as well as uses of different environments. Regarding *measured quantities*, they found a number of measures, ranging from analytical (in fact nonmeasured, such as time complexity) to empirical (such as accuracy and robustness).

To summarize, the context in which experimental approaches in computing are discussed is extremely broad. Right or wrong, experimentation terminology is by no means used in the same way it is used in, for instance, physics [81–83], biology [84], or chemistry. There are various views on the role of computing regarding experiments, there is a diversity of opinions on methods applicable, there are various examples of appropriate subjects and topics, and there are many existing analyses of experimentation in computing. However, although there are many advocates of experimentation in computing, various critical viewpoints can also be found in the literature.

### 2.2. Critique of Experimentation

Although the general atmosphere in disciplinary debates of computing has become positive towards experimental computer science, the identity of the field is still in a state of flux, and there is a notable history of critical views towards experiments and experimentation language in computing. Some critics argued that the role or the nature of experiments differs between computing and natural sciences [85, 86]. Others disputed the centrality of experiments in computing [87]. Yet others claimed that in computing experiments are not done right or are not articulated right [28, 29].

The mathematical reductionists, for one, had reservations about experimentation in computing. In his famous argument for programming as a mathematical activity, Hoare [19] complained that, because computers and programs are not constructed with mathematical rigor, the only way of finding out what they do is by experiment. He wrote that such experiments in computing certainly are not mathematics, and that because their findings often can not be generalized, “*unfortunately, they are not even science*” [19]. Hoare's answer at the time was to rigorously prove that a system will work as planned. Fletcher [87] criticized some authors' preoccupation with experimentation and noted that without the theoretical idea of Turing equivalence of all computers there would be no academic discipline of computing but just eclectic knowledge about particular machines. Many others who advocated variants of “mathematical” or “axiomatic” approaches to computing never made their stance towards experiments clear (e.g., [16]).

The second source of objections was concerned with the differences between experiment in natural sciences and in computing. Emphasizing the view that computing is a constructive discipline, Hartmanis [85] argued that experimentation in computer science is different from the natural sciences, as it focuses “*more on the how than the what*.” He wrote that whereas advancements in natural sciences are documented by dramatic experiments, in computer science—which Hartmanis [88] called the “*engineering of mathematics*”—advancements are documented by dramatic demonstrations. The role of experiments in computing, according to Hartmanis and Lin [86], is to uncover practical issues with theoretical work instead of proving those theories wrong—quite a different view compared to an idealized view of the role of experiments in science (as described in, for instance, the old falsificationist, hypothetico-deductive, and deductive-nomological models of science [89–91].)

Hartmanis [92] claimed that there are three differences between natural sciences and computing: in computing theories do not compete with each other as explanations of the fundamental nature of information; in computing anomalies in experimental results do not lead to revision of theories, and in computing there is no history of critical experiments that decide between the validity of competing theories. Hartmanis' [86, 92] views faced immediate criticism. Loui [93] responded that, instead of calling computing a new species among sciences, it would be more appropriate to call computer science a new species of engineering. Stewart [94] responded by writing that computer scientists should strive to make computer science similar to the natural sciences. Dijkstra [95] responded that it is ridiculous to support computer science and engineering as a “*laboratory discipline* (*i.e., with both theoretical and experimental components*)” if the material taught in computing has a half-life of five years. Finally, even if one accepted the controversial claim that computing has no history of critical experiments that decide between theories, there surely is a history of critical demonstrations that have decided between competing techniques and guided technical development efforts.

The third common type of objection was concerned with the artificial nature of data and subject matter of computing. McKee [96] noted that in natural sciences research is based on observations (data), which scientists can explain, predict, and replicate. In the field of computing, McKee continued that there is no data beyond the computer and programs, which behave exactly as they were designed to behave. In a similar manner, also Brooks [57] argued that computer science is not a science but a synthetic, engineering discipline. The role of experimentation in a synthetic discipline is different from its role in natural sciences (see [86, 97]).

The fourth common objection was concerned with terminology. The careless use of experimental terminology—not experiments per se—has been criticized by various authors (e.g., [60, 79]). A meta-analysis by Zelkowitz and Wallace [28, 29] revealed that terms “experiment” and “effective” were often used loosely or ambiguously. The authors wrote, “*Researchers write papers that explain some new technology; then they perform “experiments” to show how effective the technology is*.” Zelkowitz and Wallace's central concern was the same as Denning's [38]. It is not science to develop something and say that it seemed to work well.

One could add a fifth objection related to the normative claims that advocates of experimentation sometimes made. Many of those authors who urged computer scientists to experiment more failed to justify *why* computer scientists should aspire to work like scientists or engineers in other fields do. One might justly ask, “If the subject matter of computer science is different from the other sciences, on what grounds should its methods be the same?” Computing is a unique field that introduces an array of novel techniques, so perhaps some room should be left for uniqueness in methodological sense, too.

In addition to the objections, Gustedt et al. [59] proposed various assumptions that may explain the lack of experimenting in computing: insufficient funding for experimenting, “missing disposability of dedicated experimental environments,” lack of appreciation of work-intensive experimental results, and lack of methods and tools. Similarly, Tichy [27] suggested eight (mis)beliefs that he believed to explain why experiments are not more popular: “Traditional scientific method is not applicable,” “The current level of experimentation is good enough,” “Experiments cost too much,” “Demonstrations will suffice,” “There's too much noise in the way,” “Experimentation will slow progress,” “Technology changes too fast,” and “You'll never get it published.” Also Denning [38] objected against three hypothetical misconceptions about experimental computer science: “It is not novel to repeat an experiment,” “mathematics is the antithesis of experiment,” and “tinkering is experimental science.”

## 3. Five Views on Experimental Computer Science

Discussions about experimental computer science, as presented in the section above, are complicated by the various uses of the terms “to experiment” (the verb), “an experiment” (the noun), “experimentation” (the noun), “experimental” (the adjective), and the myriad derivatives of those words. The confusion was visible already in the “rejuvenating” report, and, while a lot of effort has been spent on clarifying the concepts (e.g., [28, 39, 45, 98]), there is still no agreement on experimentation terminology. This chapter presents five different uses of the term “experiment,” each relatively common in the computing literature. It should be noted that this chapter passes no judgment on “correct” uses of experimentation terminology; it only describes how it has been used in the literature.

### 3.1. Feasibility Experiment

The first and loosest use of the term “experiment” can be found in many texts that report and describe new techniques and tools. Typically, in those texts, it is not known if task *t* can be automated efficiently, reliably, feasibly, cost-efficiently, or by meeting some other simple criterion. A demonstration of experimental (novel, untested, and newly implemented) technology shows that it can indeed be done. Including the terms “demonstration” and “experimental” in the same sentence may sound like a forced marriage of two incompatible concepts, but in the computing literature “experiment” is indeed sometimes used nearly synonymously with “demonstration,” “proof of concept,” or “feasibility proof” as the following examples demonstrate.

Hartmanis and Lin [86, pages 213-214] wrote that in computer science and engineering theories develop over years of practice, with “*experiments largely establishing the feasibility of new systems*.” Plaice [99] wrote, in *ACM Computing Surveys*, that the development of large software systems exemplifies experimentation in computer science—“*and experimentation is the correct word, because we often have no idea what these tools will offer until they are actually used*.” He continued to describe that what constitutes an experiment is that a scientist “*carefully defines what must be done and then carefully sets out to do it*.” Feitelson [79] identified the “demonstration of feasibility” view as one of the three common views to experimental computer science. Feitelson also noted that the “demonstration of feasibility” experiments in applied computer science are largely divorced from theoretical computer science [79].

The *ACM FCRC Workshop on Experimental Computer Science* (http://people.csail.mit.edu/rudolph/expcs.pdf (retrieved January 30, 2013)) involved “experimental engineering” that produces new “*techniques, insights, and understanding that come from building and using computer systems*.” Hartmanis [85], though, wanted to make the difference between experiments and demonstrations explicit, calling for computing researchers to acknowledge the central role of demonstrations in the discipline. In their description of experimental computer science Basili and Zelkowitz [60], too, criticized the “demonstration” view of experimentation in computing: “*experimentation generally means the ability to build a tool or system—more an existence proof than experiment*.”

### 3.2. Trial Experiment

The second use of the term “experiment” in computing goes further than demonstrations of feasibility. The *trial experiment* evaluates various aspects of the system using some predefined set of variables. Typically, in those studies, it is not known how well a new system *s* meets its specifications or how well it performs. A trial (or test, or experiment) is designed to evaluate (or test, or experiment with) the qualities of the system *s*. Those tests are often laboratory based but can also be conducted in the actual context of use with various limitations.

Of Gustedt et al.'s [59] four-way categorization of experiments (*in situ* experiments, emulation, benchmarking, and simulation), the ones that permit the most abstraction—emulation, simulation, and benchmarking—fall into the trial experiment category. Emulation runs a real application in a model environment, simulation runs a model (limited functionality) application in a model environment, and benchmarking evaluates a model application in a real environment [59]. Similar “toy-versus-real” distinctions are made in descriptions of experimentation in software engineering [100].

McCracken et al. [41] wrote that experimental research is about “*not only the construction of new kinds of computers and software systems, but also the measurement and testing*” of those systems. Furthermore, trial experiments are not a privilege of the applied side of computing. Glass [47] proposed that formal theory needs to be validated by experiments, and Fletcher [87] wrote that theoretical computer scientists may “*resort to trial runs because the problem is mathematically intractable*.” Many types of validation of computational models of phenomena fall under trial experiments.

### 3.3. Field Experiment

A third common use of the term “experiment” is similar to trial experiments in that it is also concerned with evaluating a system's performance against some set of measures. However, the *field experiment* takes the system out of the laboratory. Typically, in those studies, it is not known how well a system fulfills its intended purpose and requirements in its sociotechnical context of use. The system is tested in a live environment and measured for things such as performance, usability attributes, or robustness. The term “field experiment” is used in, for instance, information systems [101], while Gustedt et al. [59] used the term “*in situ* experiments”: real applications executed at the real scale using real hardware.

The experimental computer science debates involve various examples of field experiments. A robot car race is an oft-used example of a field experiment, or “*experimentation under real-world conditions*” [58]. In the DARPA Grand Challenge, driverless vehicles compete with each other in finding their way through various types of environments. A common downside to the field experiment is diminished reproducibility that is brought about by the large number of variables and limited control in live environments. Yet, as they are often quasi-experiments or limited-control experiments, field experiments offer more control than case studies or surveys do [101].

### 3.4. Comparison Experiment

A fourth common use of the term “experiment” refers to comparison between solutions. Many branches of computing research are concerned with looking for the “best” solution for a specific problem [87] or developing a new way of doing things “better” in one way or another. Typically, in reports of those studies, it is not known if (or rather, “not shown that”) system *A* outperforms system *B* with data set *d* and parameters *p*. An experiment is set up to measure and compare *A*(*d*, *p*) and *B*(*d*, *p*), and the report shows that the new system beats its predecessors in terms of a set of criteria *C*. Johnson [10] called that type of experimental analysis “horse race papers.” Fletcher [87] argued that many brands of experimental computer science are most applicable to that type of research (Fletcher referred to [45, 47]).

However, although comparison experiments seem “objective” in many ways, they are, in fact, susceptible to bias in a number of ways [79, 102]. It has been noted that often such experiments do not follow the standard precautions against experimenter bias, such as the blinding principle [87]. The researcher should not be able to choose *B*, *d*, *C*, or *p* favorably for his or her own system *A*. Zelkowitz and Wallace [28, 29] argued that “*All too often the experiment is a weak example favoring the proposed technology over alternatives*.” There again, many fields of computing have introduced standard tests, input data, and expected outputs, against which competing solutions can be compared (e.g., [103]).

### 3.5. Controlled Experiment

A fifth common use of the term “experiment” refers to the *controlled experiment*. The controlled experiment is the gold standard of scientific research in many fields of science—especially when researchers aim at eliminating confounding causes—and it typically enables generalization and prediction. There are numerous uses for the controlled experiment setup; for instance, it is often used for situations where it is not known if two or more variables are associated, or if *x* causes *y*.

In many arguments for experimental computer science, by “experiment” the author explicitly or implicitly means “controlled experiment” but not always for the same reasons. Peisert [104] advocated controlled experiments for research on computer security, and their vision was that it promotes increased generalizability and better justified claims about products. Morrison and Snodgrass [71] wanted to see more generalizable results in software development. Schorr [105] argued that software and systems, with their increased user interaction, have grown too large for other kinds of methods but controlled experiments. Curtis [106] and Pfleeger [107] emphasized the role of controlled experiments in software engineering due to their potential for probabilistic knowledge about causality and increased confidence about what exactly in technical interventions caused the change. Feitelson [108] promoted evaluations under controlled conditions for all applied computer science.

## 4. Discussion

Experiments played a central part in the development of modern science, and over the centuries experiments also evolved. In modern science experiments play many roles; often in relation to theory but also independent of theory [74]. In scientific practice, the relationship between theory and experiments has always been fluid, and the many faces of experiment benefit scientific investigation in different ways at different stages of research [82]. Different fields employ experiment in different ways, and the fit between experiment, apparatus, and theory varies between disciplines [75].

The spectrum of experiments is fully visible in computing fields. The breakthroughs in computing happened at a junction of various fields, such as mathematical logic, electrical engineering, and materials science. Since the birth of the stored-program paradigm, computing has adopted methods from an even broader variety of fields. As the disciplines that gave birth to computing each have reserved a very different role for experiments, it is unsurprising that the computing literature uses experimentation terminology in a variety of ways. Sometimes the term refers to empirical research in general, sometimes to evaluation strategies, sometimes to proofs of concept, and sometimes to controlled experiments. The philosophy of experiment reveals some diversity of experimental terminology in fields other than computing, too.

The role of experiments in computing disciplines has been highly debated since the “rejuvenating experimental computer science” report in 1979. A large number of viewpoints to experimental computer science have advocated a variety of views to experiments, each with their own aims, methods, and assumptions. Experiment terminology also played a key rhetorical role in debates about the future directions of computing as a discipline. As experiments are historically central to sciences, in visions of computing as a discipline it is less risky to adopt and redefine the term “experiment” than to ignore it. The ambiguity of methodology terminology in computing parallels the situation in the philosophy of science, where experiments remained an unopened black box until the 1980s [109].

The disciplinary understanding of computing requires a naturalistic view into experiments in the field. There surely is a place for the many normative arguments on experiments in computing that have been structured around idealized views of science and the experiment. But there are also good reasons to challenge the idealized and received views of experiments. How scientists experiment has changed greatly since the days of Galileo and Bacon, as has the role of experiments in the philosophy of science. The form and function of experiments have never been rigid. The experiment has never been a mere judge between right and wrong theories. Experiment is a multidimensional phenomenon, and it is important that those dimensions are appropriately analyzed and discussed in computing, too. Also, insofar as experimentation language in computing needs clarification, it is of great help to understand the different ways in which experiments have been conceived in computing.

Methodological surveys and meta-analyses of computing research have already revealed a great diversity of views concerning empirical methods in computing, as well as what is called “experiments” in computing. Many of those views are similar to the epistemological strategies of researchers identified in the philosophy of experiment [81, 82]. Also representing and intervening—the two new characteristics of experimentation in modern science [110]—are at the very heart of modern computing, but their manifestations in computing deserve deeper analysis, especially with the age of simulation and virtual experiments.

Perhaps the use of experimentation terminology in computing should be made stricter and brought in line with some strict definitions of experimental science. Or perhaps our terminology needs to reflect what is really going on in computing and other disciplines. Either way, it is a matter of disciplinary self-understanding to take computing seriously, in its own right, and to study the discipline of computing from a nonidealized, naturalistic viewpoint. This short survey presents five faces of experiments in computing—feasibility experiment, trial, field experiment, comparison, and controlled experiment. There is a lot more to experiments in computing than what meets the eye, and we believe that their study can benefit both computing as a discipline and our general understanding of experiments in science.

## Figures and Tables

**Table 1:** Analogy between the scientific method, Colburn's [43] “solution engineering,” and Bartley's [44] view of debugging.

The Scientific method	Solution engineering	Debugging
Formulate a *hypothesis* for explaining a phenomenon	Formulate an *algorithm* for solving a problem	Make a *guess* as to what causes an identified bug

Test the hypothesis by conducting an *experiment *	Test the algorithm by writing and running a *program *	Test the guess by, for instance, tracing the program states

*Confirm or disconfirm* the hypothesis by evaluating the results of the experiment	*Accept or reject* the algorithm by evaluating the results of running the program	*Accept or reject* the guess by evaluating the program states
